# Creation of an artificial intelligence model for intubation difficulty classification by deep learning (convolutional neural network) using face images: an observational study

**DOI:** 10.1186/s40560-021-00551-x

**Published:** 2021-05-06

**Authors:** Tatsuya Hayasaka, Kazuharu Kawano, Kazuki Kurihara, Hiroto Suzuki, Masaki Nakane, Kaneyuki Kawamae

**Affiliations:** 1 Department of Anesthesiology, Yamagata University Hospital, Yamagata City, Japan; 2 Department of Medicine, Yamagata University School of Medicine, Yamagata City, Japan; 3 Critical Care Center, Yamagata University Hospital, Yamagata City, Japan; 4 Department of Emergency and Critical Care Medicine, Yamagata University Hospital, Yamagata City, Japan

**Keywords:** Tracheal intubation, Intubation difficulty, AI, Activation heat map

## Abstract

**Background:**

Tracheal intubation is the gold standard for securing the airway, and it is not uncommon to encounter intubation difficulties in intensive care units and emergency rooms. Currently, there is a need for an objective measure to assess intubation difficulties in emergency situations by physicians, residents, and paramedics who are unfamiliar with tracheal intubation. Artificial intelligence (AI) is currently used in medical imaging owing to advanced performance. We aimed to create an AI model to classify intubation difficulties from the patient’s facial image using a convolutional neural network (CNN), which links the facial image with the actual difficulty of intubation.

**Methods:**

Patients scheduled for surgery at Yamagata University Hospital between April and August 2020 were enrolled. Patients who underwent surgery with altered facial appearance, surgery with altered range of motion in the neck, or intubation performed by a physician with less than 3 years of anesthesia experience were excluded. Sixteen different facial images were obtained from the patients since the day after surgery. All images were judged as “Easy”/“Difficult” by an anesthesiologist, and an AI classification model was created using deep learning by linking the patient’s facial image and the intubation difficulty. Receiver operating characteristic curves of actual intubation difficulty and AI model were developed, and sensitivity, specificity, and area under the curve (AUC) were calculated; median AUC was used as the result. Class activation heat maps were used to visualize how the AI model classifies intubation difficulties.

**Results:**

The best AI model for classifying intubation difficulties from 16 different images was generated in the supine-side-closed mouth-base position. The accuracy was 80.5%; sensitivity, 81.8%; specificity, 83.3%; AUC, 0.864; and 95% confidence interval, [0.731-0.969], indicating that the class activation heat map was concentrated around the neck regardless of the background; the AI model recognized facial contours and identified intubation difficulties.

**Conclusion:**

This is the first study to apply deep learning (CNN) to classify intubation difficulties using an AI model. We could create an AI model with an AUC of 0.864. Our AI model may be useful for tracheal intubation performed by inexperienced medical staff in emergency situations or under general anesthesia.

## Background

It is not uncommon to encounter difficult intubation in intensive care units and emergency rooms. In addition, emergency tracheal intubation also occurs in general wards and emergency settings, and physicians and residents who are not familiar with tracheal intubation may be asked to perform it [1, 2]. There have also been cases related to out-of-hospital cardiac arrest in which paramedics have been asked to perform tracheal intubation at the scene. Intubation difficulty occurs in 5–27% of cases, and guidelines have been established to address this difficulty [3–5]. However, Rosenstock indicated that despite having guidelines for difficult intubations, recalling and following them is challenging when an effective method needs to be chosen for urgent airway clearance [6]. Chest compression needs to be interrupted during the intubation procedures in CPR, and a failure of initial tracheal intubation reduces the ability to achieve a return of spontaneous circulation in patients experiencing cardiac arrest, while likewise increasing the occurrence of adverse events such as hypoxemia and aspiration [7, 8]. In addition, mechanical damage caused by frequent intubations can lead to visual field defects such as laryngeal edema and hemorrhage, thereby complicating intubations, sustaining the inability to ventilate, and worsening the patient’s condition. Therefore, in emergency situations, it is particularly important to immediately request for the technical assistance of an experienced emergency airway management physician, rather than continuing the efforts to intubate the patient with intubation difficulty. Based on this concept, we believe that the clinical strategy to quickly and objectively determine whether the patient has intubation difficulty is crucial in emergency airway management. In addition, even skilled anesthesiologists struggle to detect intubation difficulty in patients who undergo general anesthesia. One of the reasons for this difficulty is the lack of a uniform index for the risk assessment of intubation difficulties [9]. The indicators currently used to assess intubation difficulty clinically include the Mallampati classification (MPC), inter-incisor gap (IIG), head and neck movements (HNM), thyromental distance (TMD), horizontal length of the mandible (HLM), buck teeth (BT), and upper lip bite test (ULBT) [10]. Among these approaches, the ULBT is the most accurate one by itself, but the area under the curve (AUC) of the receiver operating characteristic curve (ROC curve) is approximately 0.70 [11–13]. Another method for assessing intubation difficulty is the modified LEMON criteria [14], which takes into account (1) external appearance, (2) distance between the incisor teeth, (3) distance between the hyoid bone and the chin, (4) airway obstruction, and (5) neck immobility. The modified LEMON criterion has a sensitivity of 85% and a specificity of 47% for the prediction of intubation difficulty by direct laryngoscopy. However, the ULBT and the modified LEMON criteria have been evaluated by skilled physicians who are familiar with airway clearance, such as anesthesiologists, intensivists, and emergency physicians. The modified LEMON criteria also include a subjective assessment of the external appearance, which is typically vague and difficult to quantify. From this viewpoint, we believe that an objective measure for assessing intubation difficulty in emergency situations is essential to reduce preventable airway crises leading to the possible sudden death of the patient.

In recent years, artificial intelligence (AI) technology has developed, and image analysis systems have continued to evolve. Among them, analytical methods based on convolutional neural network (CNN) have been growing [15, 16]. The CNN-based methods have been applied in the medical field, and AI models have been created to locate intubation tubes in patients who undergo intubation based on findings from chest X-ray images and to diagnose heart failure from chest X-ray images [17, 18]. We hypothesized that this CNN could be used to discriminate the presence or absence of intubation difficulty using a patient’s facial image. If the presence of intubation difficulty can be determined in advance, then the patient’s treatment can be shifted to an anesthesiologist or emergency physician without aggravating the patient’s condition by unreasonable intubation techniques.

This study aimed to create an AI model to classify intubation difficulty using deep learning (CNN), which connects the face image of a surgical patient and the actual difficulty of intubation.

## Methods

This was an observational study of patients who received general anesthesia and were scheduled for surgery at Yamagata University Hospital from April 10, 2020 (the start date of UMIN enrollment: UMIN000040123), through August 31, 2020. Written informed consent was obtained from each patient.

**Fig. 1:**
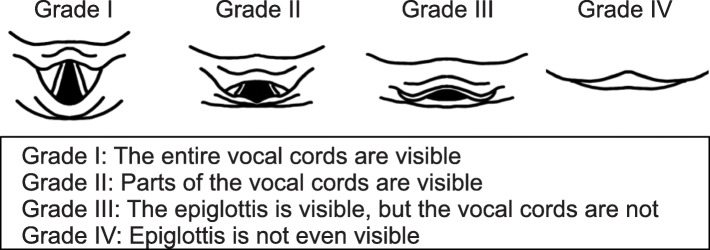
Cormack-Lehane classification. Grade I shows the entire glottis, grade II shows a part of the glottis, grade III shows the epiglottis but not the vocal cords, and grade IV shows the epiglottis is invisible

**Fig. 2:**
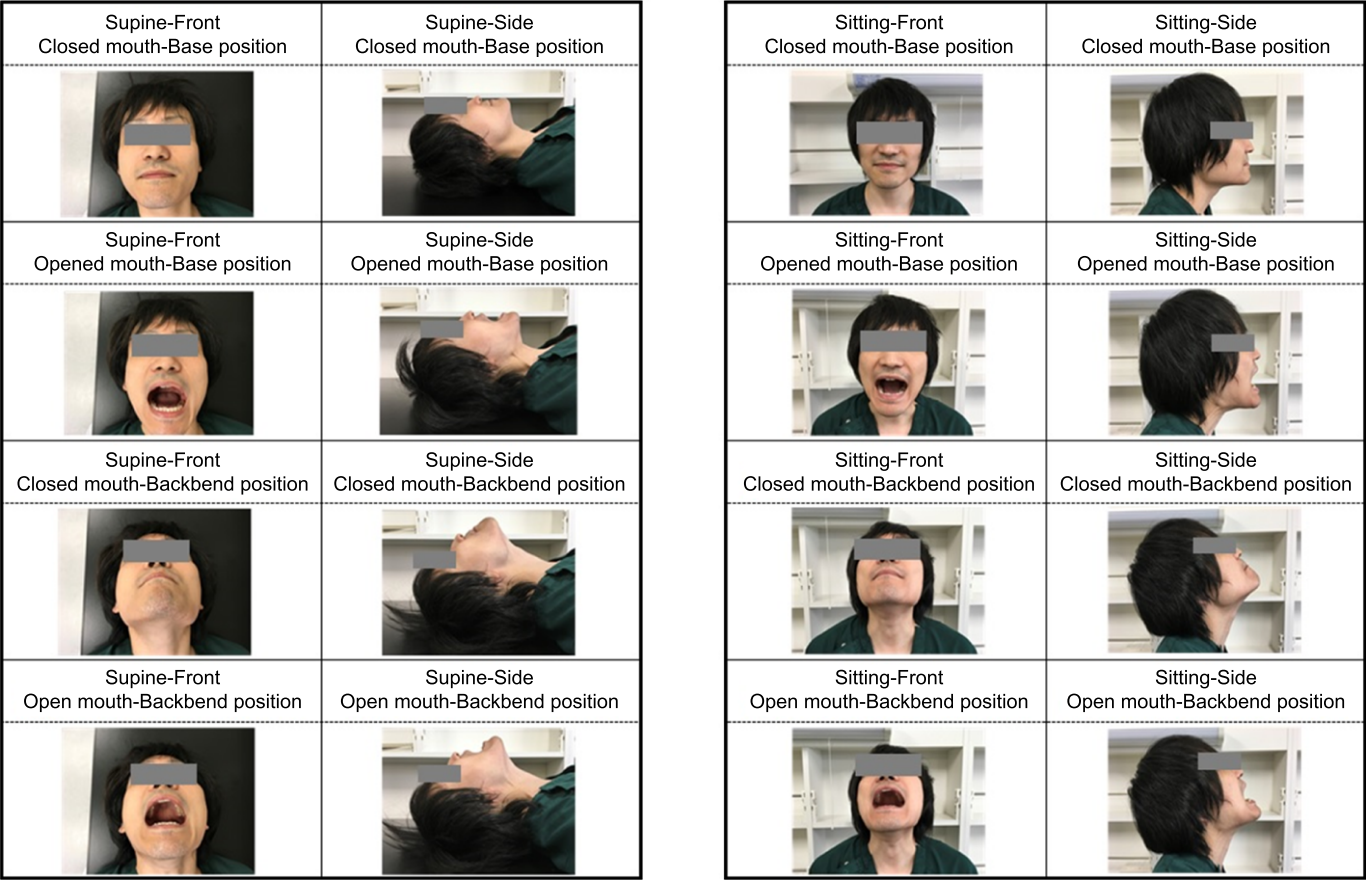
Patient face image (author’s own). Patient’s face (author’s own images). Eight patterns were captured in each of the supine and sitting positions for a total of 16 patterns

The exclusion criteria were patients younger than 20 years of age, patients who had undergone surgery with altered facial appearance (neurosurgery, heart surgery, nasal surgery, dentistry, and ophthalmology), patients who had undergone surgery with altered range of motion in the neck (thyroid, cervical spine, and esophageal surgery), and patients who underwent intubation by a physician with less than 3 years of anesthesia experience [19]. Patients whose physicians did not use the Macintosh laryngoscope at the time of initial intubation were excluded. Patients intubated with other devices, managed with supraglottic airway devices, with dementia or inability to follow instructed movements, with psychiatric disorders, and unable to participate in this study because of participation in other studies were excluded. After induction of general anesthesia, the anesthesiologist performed tracheal intubation using a Macintosh laryngoscope, and the Cormack–Lehane classification was evaluated and documented in the medical records. If the Cormack–Lehane classification was not reported in the medical records, then the author confirmed the patient’s Cormack–Lehane classification directly with the anesthesiologist. The definition of the Cormack–Lehane classification (Fig. 1) indicates the visibility of the glottis during tracheal intubation with the Macintosh laryngoscope. Grade I indicates that the entire vocal cords are visible. Grade II indicates that only parts of the vocal cords are visible, grade III that the epiglottis is visible but the vocal cords are not, and grade IV that the epiglottis is invisible [20]. In the present study, the Cormack–Lehane classification assessment was made at the time point when no special operations such as the BURP method (Backward, Upward, and Rightward Pressure) or ramp position were performed [21, 22]. The author collected information about the patient’s demographics such as age, sex, body mass index, comorbidities, MPC, IIG, HNM, TMD, HLM, BT, and ULBT from the patient the next day of the operation, and took facial images in 16 different body positions (Fig. 2). All these images were saved in JPEG format and resized to 512px × 512px to reduce excessive feature and computational complexity. The definition of intubation difficulty in this study was “Cormack–Lehane Classification grade III or higher.” All images were labeled easy and difficult; Cormack–Lehane classification grades I and II were labeled as the non-intubated difficult group (easy group), and Cormack classification grades III and IV were labeled as the intubated difficult group (difficult group) [23].

Of the obtained images, 80% were used as training data and the remaining 20% were used as test data for inference evaluation. The training data was expanded to avoid overlearning of the model. In doing so, we corrected for the bias in the number of cases between the easy and difficult groups by performing data expansion. For data expansion, we used the ImageDataGenerator class of the deep learning library Keras to expand and reduce the training data from 0.7 to 1.3 times.

**Fig. 3:**
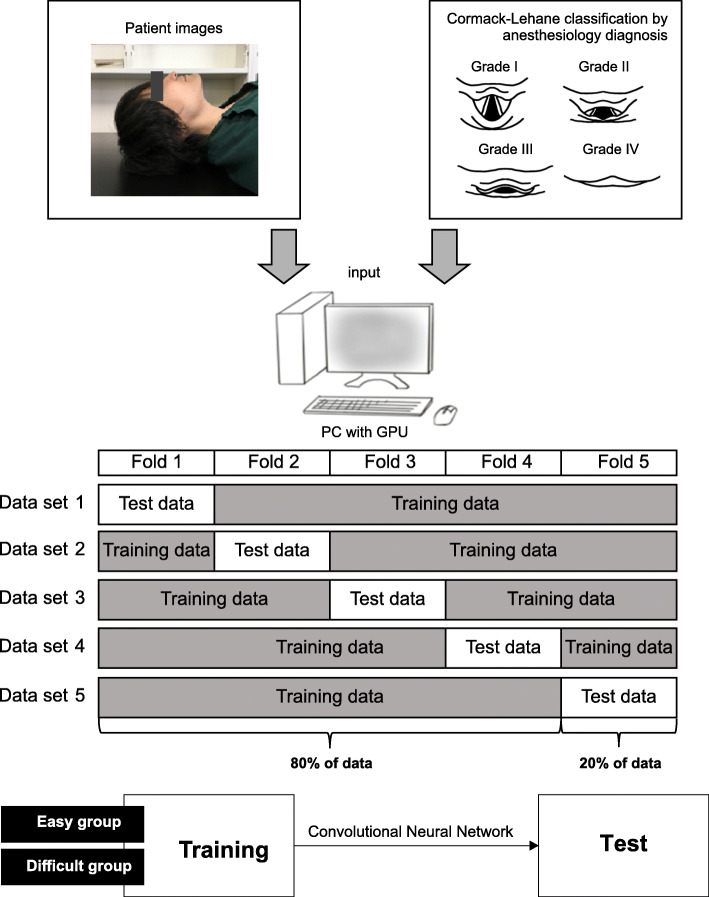
Overall view of AI model creation (author’s own). This figure shows the process of creating an AI model. To reduce the effect of data bias caused by randomly splitting the training data and test data, we split the training data from the test data by performing fivefold cross-validation and prepared five data sets

**Fig. 4:**
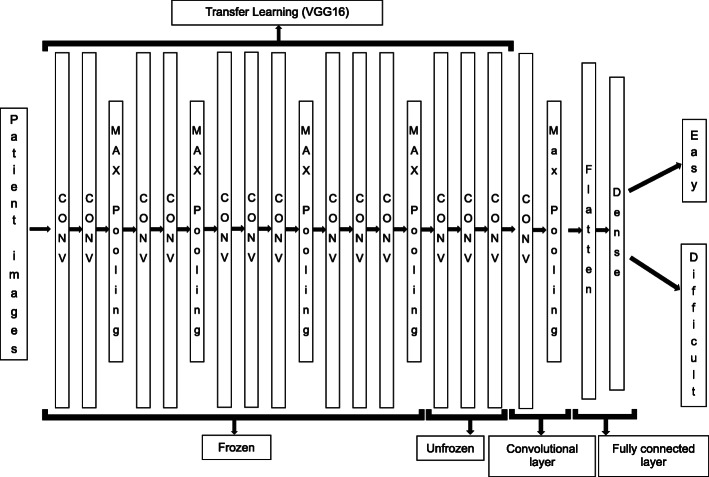
Overview of the whole model. Model generation was performed by way of a 13-layer convolutional model obtained from VGG16, adding one layer of convolution to that model

The model generation process in this study is shown in Fig. 3, and the overall model of the CNN is shown in Fig. 4. We used two methods of deep learning, transfer learning and fine tuning. Transfer learning is a deep learning technique that improves the accuracy of the AI model by incorporating a trained model created using a large data set into the model to be created [24, 25]. By using transfer learning, we can obtain a high classification accuracy of the AI model we want to create with few images because the trained model extracts good features. In this study, we used a trained model called VGG16, which is trained from 14 million images and comprises 16 layers: 13 convolutional layers and 3 fully connected layers. We also used fine tuning, which classifies the final output as easy/difficult depending on the patient face images acquired in this study [26]. The model in this study was created by adding one convolution layer to the 13 convolution layers obtained from VGG16, and the output was whether the input image belonged to easy/difficult or not. After training the model, the accuracy of predicting intubation difficulty was verified using a pre-segmented image dataset (test data) for inference evaluation. We applied quadratic cross-entropy as a loss of function and Adam as the optimization method, and the machine trained the model with 10-30 epochs and a batch size of 16-32. The evaluation metrics were the accuracy of the test data, sensitivity, specificity, and AUC calculated from the ROC curve. After the AI models were produced, the evaluation domain of the models was visualized with a gradient class activation map (Grad-CAM) using the image dataset for inference evaluation [27].

The class activation heat map is a two-dimensional image created by calculating the importance of each region based on the results of easy/difficult classification. The red and yellow areas on the heat map indicate the areas that the AI model considered important for the easy/difficult classification. The RGB values (red, green, and blue values) of each pixel in the class activation heat map of the image for inference evaluation were combined and averaged to create a single image (RGB average image) for the easy and difficult groups, respectively.

To reduce the influence of data bias caused by randomly dividing the image data for training and inference evaluation, we performed fivefold cross-validation. In addition, we used the stratified *k* fold to avoid any bias in the distribution of the easy and difficult groups when creating the five data sets. We trained and evaluated the model on each dataset and calculated the AUC of each. The median of the AUCs is shown as the result for each image model.

Keras, ver 2.24, was used as a deep learning library, and the 2019 version of Visual Studio Code from Microsoft was used as the development environment. In addition, the analysis hardware used was Intel Core i7 CPU, NVIDIA GeForce RTX 2080 SUPER 8GB GPU, and Microsoft Windows 10 Home OS. EZR, version 1.41, was used for all statistical analyses, and the results were expressed as mean ± standard deviation and numbers (percentages). ROC curves were generated from the constructed model, and the presence or absence of actual intubation difficulties, accuracy, sensitivity, specificity, and AUC were calculated. The constructed model had sufficient diagnostic capability when AUC >0.700 and the lower limit of 95% confidence interval (CI) being >0.500.

## Results

**Fig. 5:**
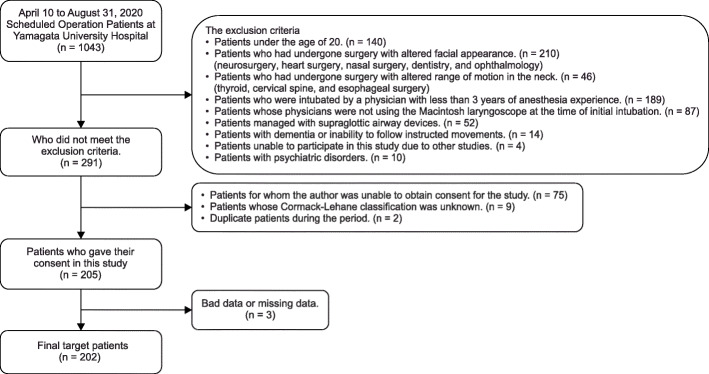
Flowchart of target patients. Informed patient consent was waived, Cormack–Lehane classification unknown, duplicate surgery patients during the period, and missing data; there were 202 patients included in the study

In total, 1043 patients were scheduled for surgery under general anesthesia from April 10, 2020 (UMIN registration start date), to August 31, 2020. Of them, 752 were excluded, 75 could not provide consent, 9 had missing data on the Cormack–Lehane classification, and 2 were duplicates. A total of 838 patients were excluded, and 205 patients were eligible. In addition, two patients with poor data (one whose facial contour could not be recognized due to the presence of hair and one whose image was out of focus) and one patient whose image was missing due to imaging equipment problems were excluded. Finally, a total of 202 patients were included in the analysis (Fig. 5). Difficulty in intubation was assessed during general anesthesia induction in 26.7% (54 of 202 patients) (Table 1). Of the 202 patients, 92 were male, and 110 were female, and their mean age was 63.9 ± 14.2 years. Patients had the American Society of Anesthesiologists Physical status (ASA PS) 1–3, with 15.8% having ASA PS 1, 67.8% having ASA PS 2, and 16.3% having ASA PS 3. The number of years of experience of anesthesiologists who intubated patients during general anesthesia was 11.2 ± 6.9 years. The surgical details in this study are shown in Table 2. Moreover, 26.7% of cases were rated as difficult to intubate (Table 1). There was a 3:1 difference in the data between easy intubation patients and difficult intubation patients. Before performing machine learning on patient face images, 20% of the total data was saved as test data. Using KFOLD1 as an example, 30 images in the easy group and 11 images in the difficult group were saved as test data (20% of the total images). The remaining 118 images in the Easy group and 43 images in the difficult group were used as training data (80% of the total images). In the training data, the easy group was expanded 3 times, and the difficult group was expanded 9 times. In the end, the easy group had 354 pieces of training data, and the difficult group had 387 pieces of training data (Table 3).

**Table 1:**
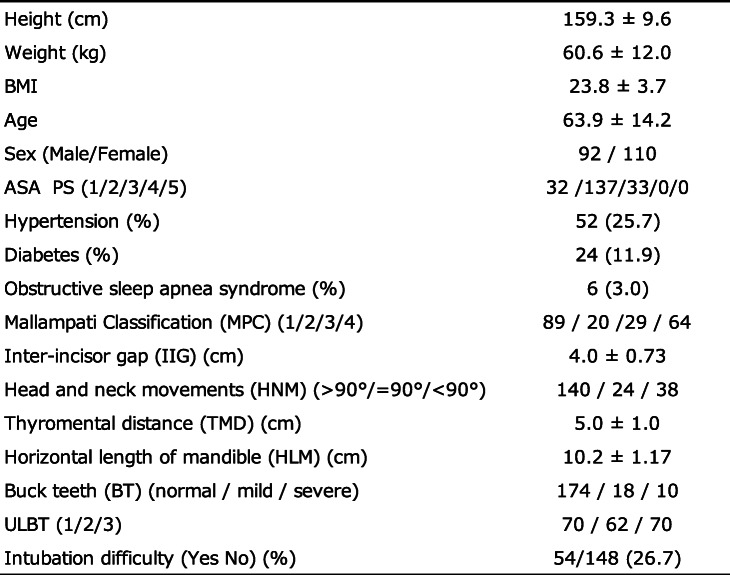
Patients’ background characteristics

The rate of intubation difficulties is 26.7%

*BMI* Body mass index, *ASA-PS* American Society of Anesthesiologists-Physical Status

**Table 2:**
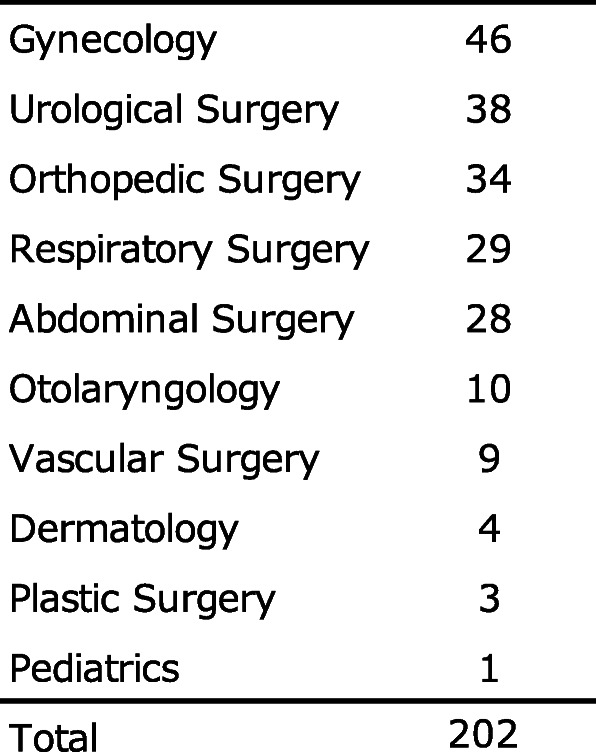
Patients’ surgical details

The number of patients by type of surgery is shown. The maximum number of patients was 48 in gynecology

**Table 3:**
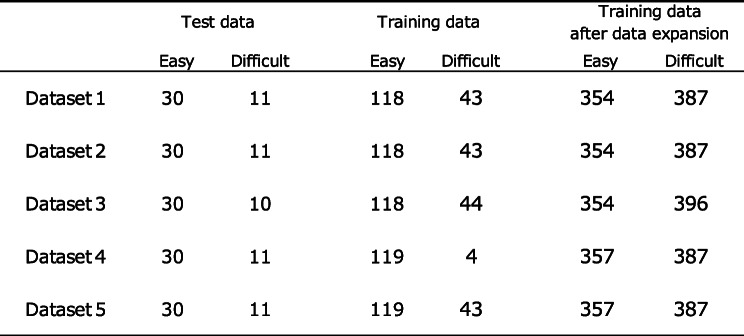
Test data and training data for each fivefold cross-validation, and training data after data expansion

The number of facial images of patients classified into the easy and difficult groups by fivefold cross-validation is shown. The number of facial images of patients in the easy group and the difficult group in the training dataset increased by 3 and 9 times, respectively

**Fig. 6:**
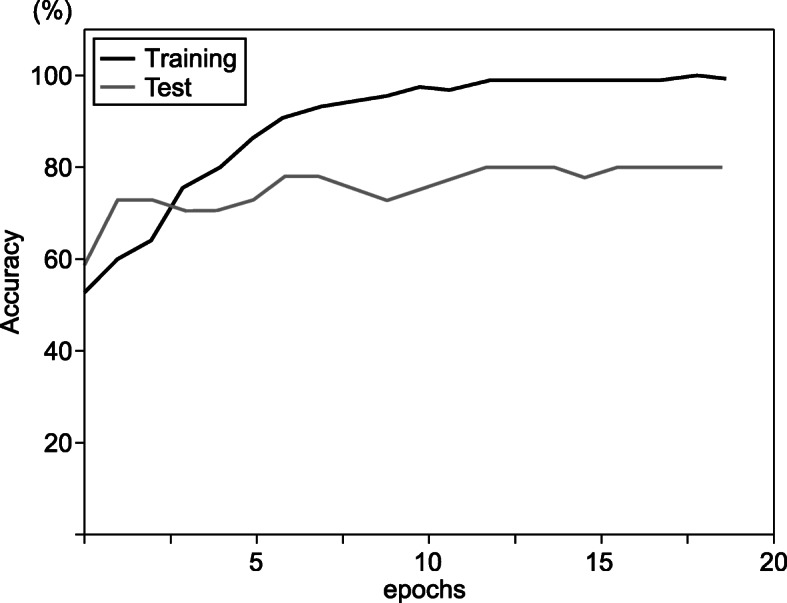
Learning curve for supine-side-closed mouth-base position. The black line (training) represents the training image data, and the gray line (test) represents the test data. The learning curve of the test data follows the learning curve of the training data, indicating that the AI model is learning properly. The AI model showed 80.5% accuracy for epochs 20

Figure 6 shows the learning curve for the supine-side-closed mouth-base position. The black line represents the training data, and the gray line represents the test data. The learning curve of the test data follows the learning curve of the training data, which indicates that the AI model is learning properly.

**Fig. 7:**
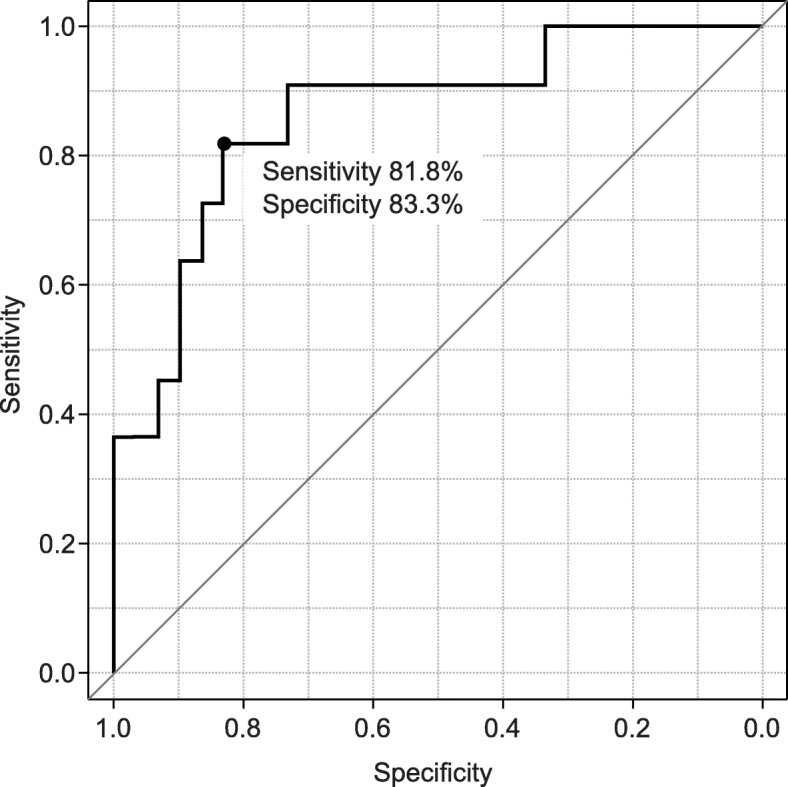
Receiver operating characteristic (ROC) curve for supine-side-closed mouth-base position. The ROC curve for discriminating intubation difficulty using face images of the supine-side-closed mouth-base position, showing an area under the curve (AUC) of 0.864, the sensitivity of 81.8%, and specificity of 83.3%

The AI model in dataset 1 in the supine-side-closed mouth-base position showed an accuracy of 80.5% at epoch 20 (Table 4). ROC curves were drawn from the AI model’s predictions to classify the actual degree of intubation difficulty and connect it with the degree of intubation difficulty obtained from patient face images. Sensitivity, specificity, and AUC were calculated (Table 5). The AI model’s AUC to classify the degree of intubation difficulty obtained from patient facial images ranged from 0.387 [0.168–0.605] to 0.864 [0.731–0.969]. The maximum AUC was 0.864 [0.731–0.969] obtained from the AI model of the supine-side-closed mouth-base position, with an accuracy, sensitivity, and specificity of 80.5%, 81.8%, and 83.3%, respectively (Fig. 7). The AI model of the supine-side-opened mouth-base position had an AUC of 0.758 [0.594–0.921], and those of the supine-side-closed mouth-backbend position had an AUC of 0.727 [0.568–0.886], which were judged to be sufficient for the diagnosis of intubation difficulty.

**Table 4:**
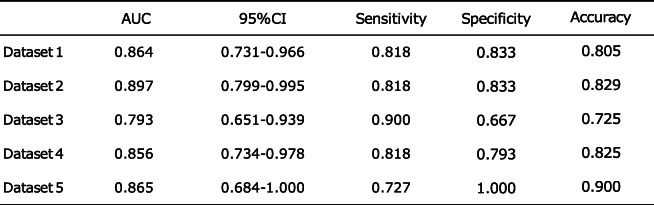
AI model accuracy of fivefold cross-validation in supine-side-closed mouth-base position

Datasets 1–5 in the supine-side-closed mouth-base position were used and divided into training data and test data

The AUC was calculated for each dataset, and the 95% confidence interval, sensitivity, specificity, and precision were shown

**Table 5:**
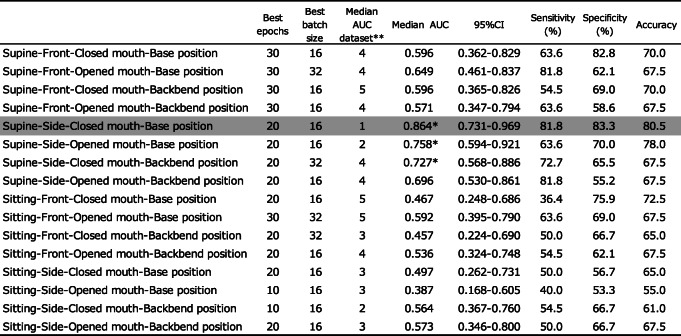
Values obtained from patient face images

The model with the best AUC value, in the range of 10–30 epochs, batch size 16–32, was created for each body position. Datasets 1–5 were used to divide the data into training data and test data. For each dataset, the AUC was calculated, the median and 95% confidence interval of the AUC for that dataset are shown, and the sensitivity, specificity, and accuracy of the median AUC are shown

^**^The name of dataset that produced the median AUC

^*^Median AUC>0.700

**Fig. 8:**
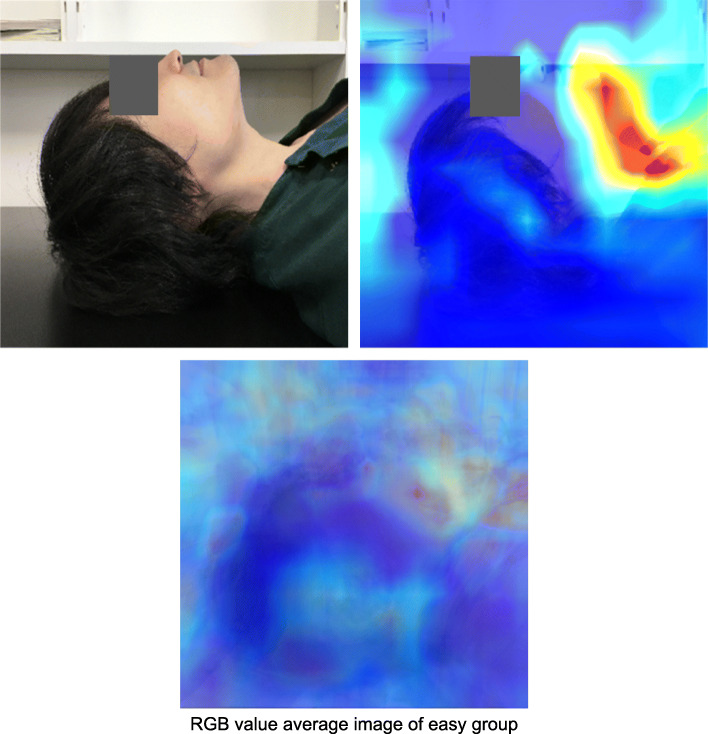
Class activation heat map by Grad-CAM of easy group in supine-side-closed mouth-base position. The heat map shows the viewpoints when the AI classifies the author’s face image (not included in the dataset) as easy to intubate, and the average RGB value image of easy intubation in the data for inference evaluation. The viewpoints that are important for the prediction of easy intubation are red and yellow in the heat map

**Fig. 9:**
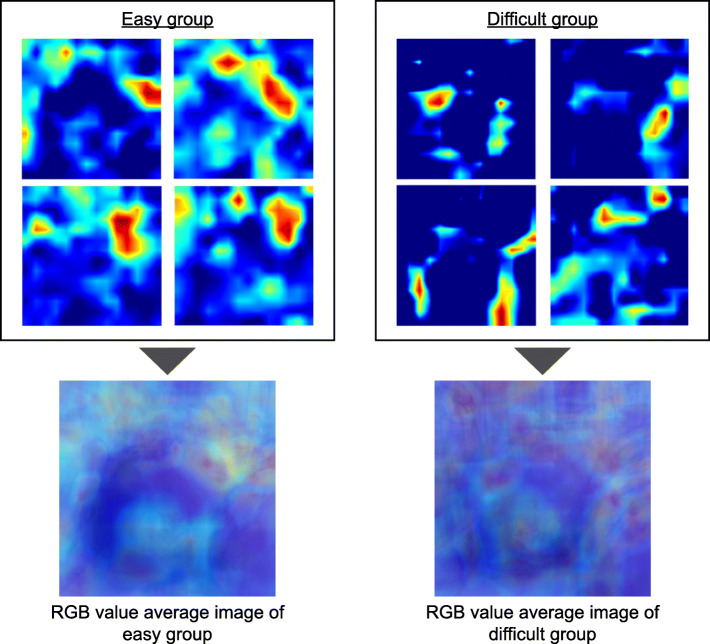
Class activation heat map by Grad-CAM for supine-side-closed mouth-base position. These are the class activation heat maps of the easy and difficult intubation groups in the supine-side-closed mouth-base position and their RGB value average image

In the class activation heat map using Grad-CAM for the supine-side-closed mouth-base position, the viewpoints tended to be concentrated in the area from the chin tip to the larynx in the images classified as easy intubation. However, the images classified as difficult did not show any concentration of viewpoints in specific areas. In the RGB-averaged images, the easy group showed a tendency for the area of interest to be concentrated from the chin tip to the larynx, while the difficult group showed a tendency for the viewpoints to be dispersed (Figs. 8, 9).

ROC curves were constructed from various predictors of intubation difficulty and the presence or absence of difficulty in actual intubation for patients in this study, and the sensitivity, specificity, and AUC were calculated (Table 6). The AUCs of the various predictors of intubation difficulty ranged from 0.558 [0.467-0.649] to 0.673 [0.595-0.750], with the Mallampati classification being the largest predictor. No single indicator was found to have sufficient diagnostic power to discriminate between the various predictors of intubation difficulties. However, the AUC of the AI model for the classification of intubation difficulty based on the images of the supine-side-closed mouth-base position was 0.864 [0.731–0.969], with an accuracy of 80.5%, a sensitivity of 81.8%, and a specificity of 83.3%, indicating that the model had sufficient diagnostic capability.

**Table 6:**
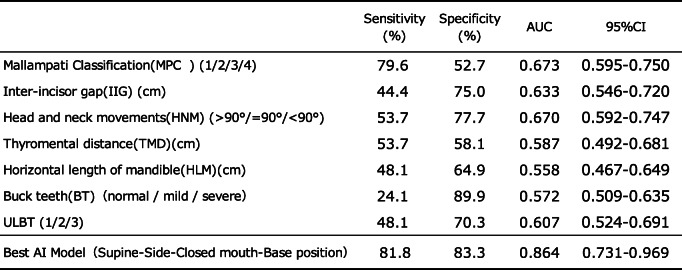
Comparison of accuracy between existing predictors of difficult intubation and the best AI model

The AUCs of the existing predictors of the difficult intubations in this study ranged from 0.558 to 0.673, and no predictor exceeded 0.700. In contrast, the best AI model produced in this study had an AUC of 0.864, which was superior to the AUC of the predictors of difficult intubation

## Discussion

In this study, in the process of creating an AI model to classify intubation difficulties by deep learning, the AI model was created using the patient’s face images taken in 16 different body positions. The best AI model for classifying intubation difficulty was taken in the supine-side-closed mouth-base position, with an AUC of 0.864 [0.731–0.969], an accuracy of 80.5%, a sensitivity of 81.8%, and specificity of 83.3% (Table 3).

In order to visualize how the AI model discriminates difficult intubation, we obtained a class activation heat map using Grad-CAM. The AI model was able to focus on the author’s neck area without concentrating on the background, indicating that the AI model recognized the contour of the face and might discriminate against intubation difficulties. The heat map showed that the area around the neck tended to be evaluated as a region of interest in the face image of a patient who was easy to intubate. The region of interest tended to be concentrated in the area from the chin tip to the larynx in the average RGB value image of the easy. This suggests that the AI model identifies easy intubations by extracting the characteristics of the neck shape. In the difficult group’s RGB-averaged images, the viewpoints tended to be dispersed, suggesting that there were multiple factors such as a small jaw and obesity in the face images of patients with difficult intubations, rather than a single cause. By increasing the number of data in the future and creating an AI model that subdivides the classification of difficult intubation, we believe that it will be possible to create a heat map of difficult intubations with extracted features. Our observations suggest that the current AI model identifies easy intubation in the easy group based on the neck.

Previous studies on the incidence of intubation difficulties report a range from 5 to 27%, compared to that of 26.7% in the present study [3, 5]. The relatively high incidence of intubation difficulty in this study may be due to the fact that the Cormack–Lehane classification was performed in the absence of the BURP method and the ramp position to provide a similar assessment to physicians who were not familiar with the application of airway assessment. The AUCs of the predictors of intubation difficulty, such as the MPC, IIG, HNM, TMD, HLM, BT, and ULBT, ranged from 0.558 [0.467–0.649] to 0.673 [0.595–0.750]. The largest predictor among them was the Mallampati classification. This result was also within the range of previous reports, and the population in this study was considered to be almost similar to those of previous studies [11–13]. The reason the AI models resulted in a better AUC than existing predictors of difficult intubation may be due to the fact that the features of multiple predictors were obtained from a single facial image. Taking the image of the supine-side-closed mouth-base position as an example, we believe that TMD, HLM, and BT are represented. Another reason is that it may have captured subjective assessments that cannot be quantified (small forehead and obesity). This may be of advantage in image analysis using CNN.

The incidence of intubation difficulties in this study was 26.7%, which led to a bias in the number of data between the easy and difficult groups. Therefore, it was difficult to create stable models using deep learning because of the bias in the allocation of training and test data. In addition, it was difficult to take patient face images at the same distance, which resulted in differences in the size of the patient’s face images. To avoid these two problems, we used the oversampling method and transfer learning to improve the accuracy. We used zoom in and out from 0.7 to 1.3 for image processing. The easy group produced three images from one image in the range of 0.7–1.3, and the difficult group produced nine images from one image in the range of 0.7–1.3. This method corrected the problems of sample number bias and distance when taking the patient’s face images. In addition, by combining transfer learning, the learning curve of the test data followed the learning curve of the training data, which was thought to avoid overfitting.

In previous studies, the Mallampati classification showed an AUC of approximately 0.60, and the ULBT showed an AUC of approximately 0.70. In this study, the results greatly exceeded the values reported in past studies due to the use of an AI model with a single facial image of the patient (image taken in the supine-side-closed mouth-base position). In addition, the modified LEMON classification used in previous studies has been shown to be highly sensitive to the assessment of intubation difficulty, but the assessment was performed by a physician familiar with the assessment of intubation difficulty. The sensitivity for predicting intubation difficulty from facial images in the supine-side-closed mouth-base position was 81.8%, suggesting that this AI model could be a skilled physician’s eye when intubating someone who is not familiar with the assessment of intubation difficulty.

The diagnosis of intubation difficulty by anesthesiologists in clinical practice is more effective in the supine position than in the seated position [28]. In this study, facial images taken only in the supine position could predict intubation difficulty. A model for predicting intubation difficulty based on face images taken in the seated position could not discriminate the presence or absence of intubation difficulty.

In a previous study, the presence or absence of intubation difficulty was discriminated by generating and quantifying facial proportions (three-way images) from a patient’s face image [29]. The study stated that the developed proportional model would take 15 min to generate a single face model, which we believe is impracticable to use in emergency situations.

This study is the first to apply deep learning (CNN) to discriminate intubation difficulty in adults. The “AI model for intubation difficulty classification using deep learning (convolutional neural network) with face images” created in this study can immediately identify intubation difficulty and can be used in emergency situations. In the future, we are planning to make “an application of the AI model for intubation difficulty classification” on the basis of this constructed model.

The limitations of the research are as follows: This study was conducted on patients who were scheduled to undergo surgery. Therefore, it is likely that the situation allows for easy intubation compared to the emergency scene or the situation of an emergency ward. Given that patients who needed devices for their difficulty in intubation (video laryngoscope) were excluded at the beginning, it is possible that some patients with difficulty in intubation may have been excluded from the study. The findings of this study are also unlikely to be applicable to pediatric intubation difficulty or congenital intubation difficulty, as the AI was trained using adult facial images [30, 31]. Older patients were often less receptive to having their faces photographed, which may have resulted in a relatively young patient population. Furthermore, this study was conducted only at Yamagata University Hospital and is an AI model produced with patient face images from a limited area.

## Conclusions

In this study, an AI model was created to classify intubation difficulty by deep learning (CNN) using face images. The AI model obtained from face images taken in the supine-side-closed mouth-base position showed the best predictive value, i.e., 80.5%. This is the first attempt to apply deep learning (CNN) to discriminate intubation difficulty. We believe that, in the future, a clinically useful model can be created with a larger number of face images in a larger area. If the AI model can predict intubation difficulty using the patient’s face image, then it can help save patients’ lives by enabling rapid requests for assistance to physicians who are familiar with emergency airway management without causing visual field defects due to unreasonable tracheal intubation.

## Data Availability

The data underlying this article will be shared on reasonable request to the corresponding author.

## Abbreviations

CPR: Cardiopulmonary resuscitation
MPC: Mallampati classification
HLM: Horizontal length of mandible
BT: Buck teeth
ULBT: Upper lip bite test
AUC: Area under the curve
AI: Artificial intelligence
CNN: Convolutional neural network
ASA PS: American Society of Anesthesiologists Physical Status
